# The Poison Squad: One Chemist’s Single-Minded Crusade for Food Safety at the Turn of the Twentieth Century

**DOI:** 10.3201/eid2607.200184

**Published:** 2020-07

**Authors:** Michael C. Bazaco

**Affiliations:** US Food and Drug Administration, College Park, Maryland, USA;; University of Maryland School of Public Health, College Park

**Keywords:** poison, chemistry, food safety

Formaldehyde made spoiled milk taste tolerable. Ground coffee was more than likely ground chicory. The yellow coating of children’s candies often contained arsenic. Ketchup was a soup of “waste products from canners, like coal-tar colors or starch paste.” Thus was the status of the foods the US public ate during the late 1800s. In The Poison Squad, author Deborah Blum tells the story of Harvey Washington Wiley and his dogged pursuit of government regulation for food safety and purity at the turn from the 19th to the 20th century (Figure).

**Figure:**
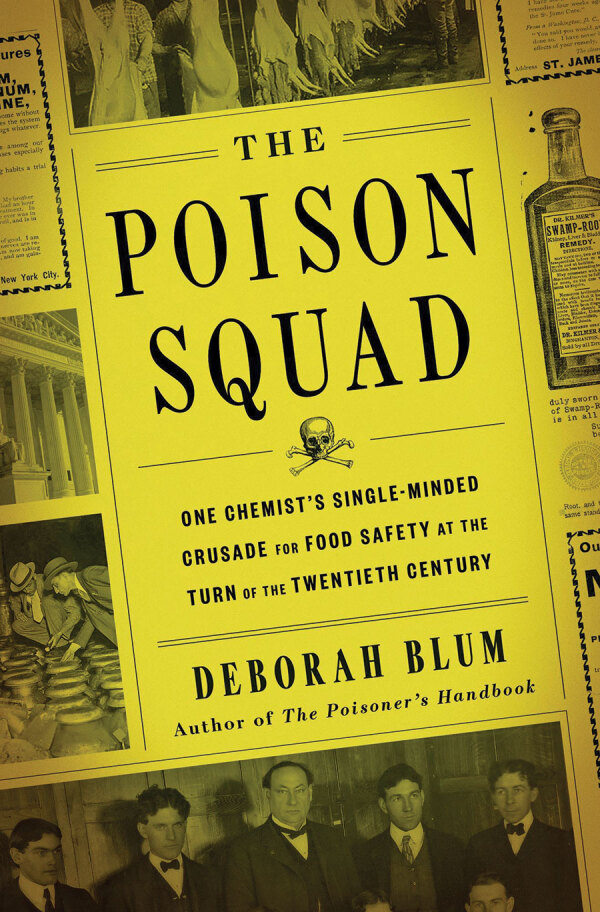
The Poison Squad: One Chemist’s Single-Minded Crusade for Food Safety at the Turn of the Twentieth Century

She first details the food industry before his work, when dangerous chemicals were used without regulation to cut, mimic, or enhance the flavor of foods with little regard for effects on human health. She then describes the initial failed attempts of food safety advocates and their efforts to focus the attention of the general public and then the US Congress on food safety. Passage of the Pure Food and Drug Act of 1906 was only the first part of Wiley’s story. Appropriately, the first section of The Poison Squad ends there.

In the second half, Blum deftly walks through the varied and complex difficulties Wiley faced in enforcing the laws mandated by the act. These difficulties included a hesitant and sometimes antagonistic boss, co-workers pitted against him, the various priorities of multiple presidents, and large and powerful food and chemical companies looking to work around the law. However, as Blum so keenly explains, Wiley was not alone in his quest. He had loyal supporters inside his own Bureau of Chemistry at the US Department of Agriculture, of course. But more important, he made allies along the way, such as Alice Lakey of the National Consumers League; colleagues at the American Medical Association; and even H.J. Heinz, founder of the food company that still bears his name. Blum does a wonderful job describing the value of these supporters to Wiley as he struggled for food purity and public health.

With intriguing anecdotes, vivid quotations, and expert attention to detail, Blum guides readers through the tedious process of moving an idea into legislation, applied regulation, and finally enforcement, and she does so with a storyteller’s touch. This captivating book relates the importance and practical impacts of Wiley’s endeavors to the life of an ordinary citizen of the time. It is equal parts informative and entertaining.

Blum’s optimistic book demonstrates that, with hard work and determination, laws can be passed to protect public health, even when opposition is fierce. It also shows that forming and nurturing partnerships with citizen groups, academic institutions, and industry leaders is key to these endeavors. Although the book does an excellent job describing Wiley’s contributions, it only briefly addresses some of the ethical issues regarding his actual “Poison Squad” studies, or the flaws in their study design. This book will educate and inspire readers who have worked in public health for decades and those who are just entering the field. It will also inform persons who work toward other public service endeavors and entertain those who simply like a good story. A Public Broadcasting Service special that closely follows this book is also available (https://www.pbs.org/wgbh/americanexperience/films/poison-squad).

